# A high-fat diet decreases GABA concentration in the frontal cortex and hippocampus of rats

**DOI:** 10.1186/s40659-016-0075-6

**Published:** 2016-02-29

**Authors:** Cuauhtemoc Sandoval-Salazar, Joel Ramírez-Emiliano, Aurora Trejo-Bahena, Cecilia I. Oviedo-Solís, Martha Silvia Solís-Ortiz

**Affiliations:** Departamento de Enfermería y Obstetricia, Universidad de Guanajuato, Celaya, GTO Mexico; Departamento de Ciencias Médicas, División de Ciencias de la Salud, Campus León, Universidad de Guanajuato, 20 de Enero 929, C.P. 37000 León, GTO Mexico; Departamento de Medicina y Nutrición, Universidad de Guanajuato, León, GTO Mexico

**Keywords:** Obesity, GABA, Frontal cortex, Hippocampus, High-fat diet

## Abstract

**Background:**

It has been proposed that the γ-aminobutyric acid (GABA) plays a key role in the regulation of food intake and body weight by controlling the excitability, plasticity and the synchronization of neuronal activity in the frontal cortex (FC). It has been also proposed that the high-fat diet (HFD) could disturb the metabolism of glutamate and consequently the GABA levels, but the mechanism is not yet clearly understood. Therefore, the aim of this study was to investigate the effect of a HFD on the GABA levels in the FC and hippocampus of rats.

**Results:**

The HFD significantly increased weight gain and blood glucose levels, whereas decreased the GABA levels in the FC and hippocampus compared with standard diet-fed rats.

**Conclusions:**

HFD decreases GABA levels in the FC and hippocampus of rat, which likely disrupts the GABAergic inhibitory processes, underlying feeding behavior.

## Background

Obesity is a serious and growing public health problem that reduces life expectancy and increases morbidity due to the development of complications, such as cardiovascular diseases, glucose intolerance, hyperinsulinemia, type 2 diabetes, dyslipidemia, and hypertension [1]. Psychological factors, genetic predisposition and eating habits can induce obesity [1]. With respect to food habits, an increased intake of diets rich in saturated fat can contribute to cognitive decline [2]. Thus, the high-fat diet (HFD)-induced obesity models have contributed significantly to the study of the pathophysiology of insulin resistance, hyperglycemia, obesity and metabolic syndrome [3], might also be helpful to understand how HFD contributes to cognitive damage [2].

GABA (γ-aminobutyric acid) has been proposed to play a key role in the cognitive choice of selecting the type, quantity and quality of food by regulating the transmission of signals between neurons in brain circuits [4]. GABA is the major inhibitory neurotransmitter in the mammalian brain and has been implicated in controlling excitability, the processing of information, plasticity, and the synchronization of neuronal activity [5]. Additionally, the involvement of GABA in eating behavior has been supported in experiments using rodent models and GABA receptor agonists and antagonists [6]. Changes in brain glucose concentration can modify the release of neurotransmitters. For instance, hypoglycemic periods inhibit GABA release in the substantia nigra and ventral tegmental area (VTA), which are areas that project to the frontal cortex (FC), and increases dopamine concentration and disinhibits the FC [7].

The prefrontal cortex (PFC) plays a role in cognitive functions such as food selection [8], and PFC neurons are activated when individuals show a preference for an appetizing food instead of a non-appetizing one [8]. Thus, in humans, it has been proposed that the disinhibition and asymmetry of the FC could induce eating disorders by increasing anxiety and appetite, resulting in the development of obesity [9]. Additionally, it has been proposed that the PFC plays a crucial role in the top-down control of behavior, especially under conflict situations when inappropriate responses need to be inhibited [10], likely by modulating GABA levels.

The PFC has connections with the medial temporal limbic system and outputs that include direct and indirect connections with the amygdala, hypothalamus and hippocampus [11].

The hippocampus is involved in the regulation of the learning and memory functions [11] and recently has received increasing attention for its potential involvement in the regulation of energy homeostasis and feeding behavior through leptin signaling, which contributes to the non-homeostatic control of food intake by suppressing the ability of contextual cues to elicit a memory for food [12]. A HFD alters glutamate metabolism and neurotransmission in the rat hippocampus, along with a significant change in glutamate uptake and a reduced synaptic efficacy [13]. Therefore, a HFD could disturb the metabolism of glutamate and consequently, GABA levels [14]. The hippocampal damage could alter body weight regulation [15], affecting processes that contribute to the control of appetite behavior [12].

Obesity reduces aptitude in learning tasks, including decision making, memory and other cognition processes in humans and animal models [8]. Most obese patients are impulsive and do not resist the consumption of foods that generate and/or maintain their obesity [16]. The effect of a HFD on GABA levels in the FC and hippocampus is not well understood, but it is likely that the disturbance of GABA levels induces the development of overweight and obesity. Therefore, the aim of this study was to investigate the effect of a HFD on GABA levels in the frontal cortex and hippocampus of rats.

The present results indicate that a HFD decreases the GABA concentration in the FC and hippocampus. These findings also suggest that a HFD might disrupt processes of inhibition and subsequently alter food intake.

## Results

### Effect of a high-fat diet on body weight gain

**Fig. 1:**
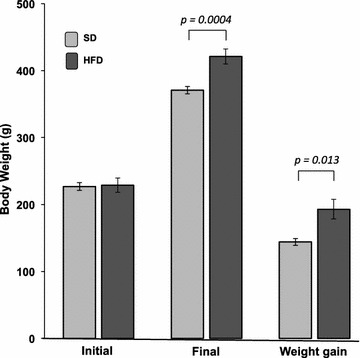
Effect of high-fat diet on body weight gain. SD, standard diet (n = 10); HFD, high-fat diet (n = 10). Data are given as the mean ± standard error of the mean (SEM)

To examine the effect of HFD on the gain of body weight, the rats were weighted at the beginning of the HFD treatment and no significant differences were observed between the SD and HDF-fed rats (*p* = 0.6), Fig. 1. At the end of the treatment, the HFD group had a significant increase in body weight compared with the SD group (*p* = 0.0004). The HFD-fed rats gained significantly more weight than SD-fed rats (*p* = 0.014).

### Effect of a high-fat diet on biochemical parameters

Table 1 shows the serum glucose, cholesterol and triglycerides levels. The glucose levels of the HFD-fed rats were significantly higher compared with the SD-fed rats (*p* = 0.001). The SD and HFD groups had similar cholesterol levels (*p* = 0.168). The consumption of a HFD did not increase triglyceride levels after eight weeks of exposure compared with the SD consumption (*p* = 0.87).

**Table 1:** Biochemical parameters of rats

	Standard diet (n = 7)	High-fat diet (n = 7)	T value	P value
Glucose (mg/dl)	89 ± 9.3	155 ± 7.5	−5.53	0.001
Cholesterol (mg/dl)	83 ± 1.41	110 ± 17.8	−1.56	0.168
Triglycerides (mg/dl)	75 ± 3.9	89 ± 5.9	−2.04	0.87

Data are given as the mean ± SEM

### A high-fat diet decreases GABA levels in the frontal cortex and hippocampus

**Fig. 2:**
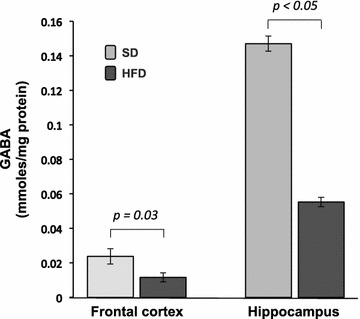
Effect of high-fat diet on GABA levels in frontal cortex and hippocampus. SD, standard diet (n = 10); HFD, high-fat diet (n = 10). Data are given as the mean ± standard error of the mean (SEM)

Figure 2 shows the GABA levels in the FC and hippocampus. The HFD-fed rats showed a significant decrease in their GABA levels in the FC compared with the SD-fed group (*p* = 0.00003), similar to the GABA levels in the hippocampus in comparison with the SD-fed rats (*p* < 0.03).

## Discussion

Hypercaloric diets can modify the GABA levels in the brain. As predicted, we found that a HFD administered to rats for eight weeks increased the body weight and blood glucose and decreased the GABA concentration in the FC and hippocampus.

The body weight gain observed in the present study is consistent with studies in animal models, suggesting that exposure to high concentrations of carbohydrates or HFD contribute to the development of overweight or obesity [3]. Fat accumulation is stimulated when nutrients are led to the adipose tissue or to other tissues in which oxidation is the immediate outcome [17]. Studies indicate that an imbalance in the activities of the glucose transporter GLUT4 and lipoprotein lipase between muscle and adipose tissue may be important in the development of obesity [18]. Therefore, it is a logical conclusion that alterations limiting lipolysis and fatty acid oxidation and stimulating lipogenesis cause or are associated with obesity [19].

In rats, a diet with a high content of carbohydrates and fat increases the blood glucose levels [19]. This diet type eventually results in moderate hyperglycemia and impaired glucose tolerance in most rat and mouse strains [20]. C57BL/6 J mice fed with a HFD had higher glucose levels [21], which is consistent with our data. Studies using hyperglycemia models have found that rats exposed to a hypercaloric diet for weeks develop changes in the blood glucose levels [22, 23]. We fed rats a HFD for eight weeks and also found increased glucose levels, but the cholesterol and triglycerides levels did not change. This may be due to the animals still having the ability to increase energy reserves without changing the blood levels of cholesterol and triglycerides. A large increase or decrease in the blood glucose level causes that the brain to respond and initiate compensatory responses to maintain glucose homeostasis [24]. Therefore, an increase in circulating glucose levels suggests that there is a disturbance in brain metabolism, which could increase the risk of developing overweight and obesity.

There is a relationship between HFD intake and neurotransmitter concentration in the rat brain [13]. For instance, saturated fats and simple carbohydrates produce feelings of gratification by activating brain circuits through changing neurotransmitters levels [25]. Moreover, glucose modulates GABA and dopamine release in the substantia nigra (SN) and VTA [7]. Additionally, GABAergic neurons in the VTA have projections to many brain regions, including the PFC, a structure involved in brain reward [7]. We found that the GABA concentration was lower in the FC of HFD-fed rats. It is likely that a HFD disturbs the inhibitory control of appetite and induces the development of obesity by modulating cerebral neurotransmitter metabolism. Thus, GABA levels in PFC are thought to play a key role in the modulation of food intake and body weight gain, although the mechanism is not yet clearly understood.

The role of GABA in the PFC for the regulation of food intake is not well understood. In glia, GABA is degraded by the enzyme GABA-transaminase through the transamination reaction of α-ketoglutarate, producing l-glutamate, which leads to the formation of succinic semialdehyde [a negative allosteric modulator of the enzyme Glutamic Acid Decarboxylase-(GAD)] [26]. A study in rats suggests that obesity may be associated with decreased GABA production due to a disturbance in the GABA-glutamate-glutamine cycle in the hippocampus [14]. We found decreased GABA concentration in the PFC of rats fed a HFD, which could be detrimental to oxidative glucose metabolism and the synthesis of neurotransmitter (i.e., glutamate and GABA). This could cause homeostatic dysregulation between neurons and astrocytes. Thus, it is likely that this homeostatic dysregulation can affect the regulation of the eating behavior by promoting an imbalance in the PFC and their connections with the reward system. The PFC receives highly processed gustatory and olfactory information along with limbic input from the amygdala and hippocampus and sends projections to the nucleus accumbens Shell, which is involved in profound hyperphagia by decreasing levels of GABA or the opiate receptor [27]. Pathological changes in vmPFC function impair the control of food intake and may facilitate eating disorders, obesity, and other disorders of appetitive motivation [28]. Food intake was no measured and even not the role of GABA is not clear in this condition. We believe that a decrease in GABA levels might result in less inhibition of food intake. For example, Le et al. 2006 compared lean men with obese men and found a less postprandial activation in the left dorsolateral prefrontal cortex [29]. This area has seen implicated in the inhibition of inappropriate behavior and differential responses of neuronal activity to food intake. This area probably may contribute to a propensity for obesity or to the difficulty in losing weight experienced by obese men. Thus, it is important to evaluate the eating behavior and other cognitive processes, which involve GABA, glutamate or dopamine.

The hippocampus is a brain structure that contains receptors for several hormonal signals, such as insulin and leptin, which may control energy balance [12]. A study in mice showed that a HFD alters glutamate metabolism and neurotransmission in the hippocampus, along with a significant change in glutamate uptake and a reduced synaptic efficacy [13]. Therefore, an HFD may disrupt the metabolism of glutamate and consequently that of GABA [14] and increase the number of reactive astrocytes and neurodegeneration [30]. Interestingly, we observed that the GABA concentration decreased in the hippocampus of HFD-fed rats compared with SD-fed rats, which suggests an alteration in the process of feeding motivation. Therefore, these results allow for a better understanding of the relationship between HFD and GABA concentration.

In our opinion, the decrease of GABA levels could increase neuronal hyperexcitability. Thus, a reduction of GABA in the hippocampus and frontal cortex could decrease the processes of inhibitory control of food intake. Nevertheless, we did not measured the food intake, but a study found that obese individuals have a hypo-functioning reward circuitry, which suggest a less inhibitory function and therefore, the food appear to be more attractive to obese subjects during hunger state [31]. It is necessary other experiments which involve eating behavior like food choices and to determine the participation of other neurotransmitters such as glutamate and dopamine.

## Conclusions

The present results showed that a HFD reduced GABA levels in the FC and hippocampus. These findings suggest that the lower neurotransmitter levels in the FC and hippocampus could impair the inhibitory processes underlying feeding behavior. The results also promote new strategies with pharmacological agonists and antagonists of GABA to understand how GABA level disturbances are involved in the brain mechanisms relating to obesity and probably imbalanced overeating.

## Methods

### Experimental animals

Twenty adult male Wistar rats that were of 2–3 months old (200–250 g of weight) were maintained in polypropylene animal cages in a temperature-controlled environment (22 ± 2 °C) and under a light–dark cycle set at 12:12 h in the University of Guanajuato Animal Facility. All of the animal procedures in this study were conducted in accordance with the National Research Council Guide for the Care and Use of Laboratory Animals and the Official Mexican Regulation for Experimentation in Animals (NOM-062-ZOO-1999).

### Treatment with a high-fat diet

The rats were randomized into two groups. The first group consisted of 10 rats fed a standard diet (Purina Rodent Chow; Purina Mexico), while the other group consisted of 10 rats fed a high-fat diet (Purina Chow, 13 % lard; Purina Mexico). The Table 2 shows the composition of the diets. The two groups had access to water and chow ad libitum for eight weeks.

**Table 2:** Comparison between the composition of the standard diet and the high fat diet

	Standard diet (%)	High fat diet (%)
Carbohydrates	58.5	48
Protein	23	29
Lipids (lard)	4.5	13
Others constituents	14	10

### Collection of blood and tissue samples

At the end of the 8 week treatment, the rats were sacrificed by cervical dislocation. Immediately, 3 ml of blood was collected directly from the heart and placed into tubes without anticoagulant to separate the serum. The tubes were centrifuged at 3000 rpm, and 500 μl aliquots of serum were stored at −20 °C. Subsequently, the FC and hippocampus were removed, dissected on ice-cold glass and stored in microtubes at −80 °C until analysis. The FC and hippocampus tissues were homogenized in Eppendorf tubes with 500 μl of 0.1 M hydrochloric acid. The homogenates were centrifuged (10,000 × g, 4 °C, for 30 min). The supernatants were stored at −20 °C until analysis of the GABA concentration.

### Determination of glucose, cholesterol and triglycerides levels

In serum, the glucose, total cholesterol and triglycerides levels were measured using a spectrophotometric method and a SPINREACT Kit (Glucose-TR, GOD-POD; Cholesterol, CHOD-POD and Triglycerides GPO-POD and Enzymatic techniques. LAB CENTER MEXICO). The data are expressed as mg/dl.

### Chemicals for GABA concentration determination

For determining the GABA concentration, the following reagents were used: (1) GABA; (2) Dns (1-dimethylamino naphthalene sulfonyl chloride), both purchased from Sigma, St. Louis, MO; (3) acetonitrile and hydrochloric acid were (from Karal SA de CV); (4) NaHCO_3_ and potassium hydroxide (from KEM Baker Analyzed, Inc. NJ USA); (5) high-performance liquid chromatography (HPLC) grade water (from the Department of Medical Science, León, Mexico); (6) an chromatographic column, Hydrosphere C18, 120A, 5 µM 150 × 4.6 mm (used for GABA separation and purchased from YMC Co., Ltd. Kyoto, Japan).

### GABA level determination

The GABA level was quantified using HPLC–UV detection and acetonitrile–water (35:65, v/v) as the mobile phase. The derivatization of GABA was performed with Dns, using modified Kang´s method [32]. Stock solutions of GABA (1 mg/ml) were prepared in HPLC water. These solutions were diluted daily to working concentrations with water and were stored at 4 °C in the dark. The Dns solution was prepared just before derivatization by dissolving 200 mg of Dns in 10 ml of acetonitrile. All of the frozen samples were thawed just before the experiment. A total of 50 µl of the sample, 50 µl of 2 M NaHCO_3_–KOH solution (pH 9.8) and 20 mg/ml of Dns in acetonitrile (50 µl) were mixed and incubated at 80 °C for 30 min in a water bath under dark conditions. Next, an aliquot (20 µl) of acetic acid was added to the tube to stop the reaction. The mixture was centrifuged at 10,000 x g for 5 min. The supernatant (150 µl) was injected into the HPLC system, and the derivatives were measured at 286 nm. The HPLC system included a GBC LC-1150 pump, a GBC 1650 Advanced Autosampler and a GBC LC1210 K UV–Vis Detector. The HPLC flow rate was 1.5 ml/min. All of the measurements were performed at room temperature.

### Determination of protein content

The tissue protein content was determined according to the bicinchoninic acid method using bovine serum albumin as the standard.

### Statistical analysis

The statistical analyses were performed with Statistics for Windows 8 (StatSoft, Inc.). Student’s *t* test was used to compare the body weight, biochemical parameters and GABA concentrations between the groups with the significance level set at *p* < 0.05. The results were expressed as the mean ± standard error of the mean (S.E.M.).
